# Built environment effects on older adults’ health mediated by leisure activities and social participation in Dong settlements of Western Hunan

**DOI:** 10.1038/s41598-026-51985-y

**Published:** 2026-05-08

**Authors:** Yuhan Luo, Xian Liu, Qingchan Wang, Mengen Gu, Yingyi Li, Wenbin Luo

**Affiliations:** 1 School of Art, Hunan University of Information Technology, Changsha, 410151 China; 2 Maanshan Teacher’s College, Maanshan, 243041 China; 3 Hunan International Economics University, Changsha, 410205 China

**Keywords:** Anthropology, Environmental social sciences, Environmental studies, Geography, Geography, Sociology

## Abstract

Against the dual backdrop of population aging and China’s Rural Revitalization Strategy, investigating the mechanisms through which the built environment in traditional settlements is associated with older adults’ health is of substantial significance. Taking traditional Dong ethnic settlements in Western Hunan as a case study, a theoretical model encompassing “objective environment-subjective perception-behavioral mediation-health outcomes” was constructed to examine the associations and pathways linking the built environment to older adults’ health, with leisure activities and social participation considered both as independent and sequential mediators. The findings revealed that: (1) objective built environment shows no direct association with older adults’ health; rather, its health-related benefits appear to be entirely mediated by leisure activities and social participation; (2) perceived built environment is not only directly associated with older adults’ health but also demonstrates indirect positive associations through leisure activities, social participation, and their sequential mediation pathways; (3) within the context of traditional “acquaintance societies,” social participation holds a central position, with leisure activities further associated with health through their relationship with social participation, thereby creating a hierarchical mechanism of association. The findings suggest that the health benefits of traditional settlements may not originate from the physical space per se, but appear to be deeply embedded in residents’ subjective environmental perceptions and sociocultural practices. This research expands the contextual scope of built environment and health research and deepens understanding of health-related behavioral mechanisms among older adults in traditional communities. The findings provide theoretical reference and empirical evidence for the development of age-friendly rural environments and the advancement of healthy aging practices.

## Introduction

Currently, global population aging is accelerating, and older adults’ health issues are receiving increasing attention. According to statistics, the global population aged 65 and above reached 761 million in 2021, with projections indicating an increase to 1.6 billion by 2050^1^. As of the end of 2024, China’s population aged 60 and above reached 303 million, accounting for 22% of the total population, with those aged 65 and above comprising 220 million, representing 15.6% of the total population. According to projections by the National Health Commission (NHC), China’s population aged 60 and above is expected to exceed 400 million by 2035, accounting for more than 30% of the total population^2^. The persistent aging of population structure will impose increasingly higher demands on social security systems, health services, and the development of livable environments. Although advances in public health and healthcare have significantly extended human life expectancy, aging populations face multiple health challenges, including physiological decline, high prevalence of chronic diseases, and psychological loneliness^3^. Residential conditions have been recognized as a critical structural determinant of health, shaping residents’ well-being through both physical and social dimensions of the living environment^4^. In recent years, an increasing number of studies have examined the potential impact of the built environment in urban communities on older adults’ health, recognizing its significant role in promoting physical activity, enhancing social interaction, and improving psychological well-being^5,6^. However, rural areas continue to face substantial deficiencies in infrastructure, public services, and spatial environments, and the health appropriateness of residential spaces for older adults in traditional settlements has long lacked systematic investigation and targeted interventions. In China, the Rural Revitalization Strategy, formally launched in 2018 as a national policy initiative, has prioritized the comprehensive improvement of rural living environments, the preservation of traditional villages, and the promotion of rural residents’ well-being^7^. Within this policy context, addressing the health needs of the rapidly growing rural older adult population has become an increasingly pressing agenda, particularly in remote ethnic minority settlements where infrastructure deficiencies and limited public health resources remain pronounced. Therefore, investigating the mechanisms through which the built environment in traditional rural settlements influences older adults’ health status (HS) carries urgent practical significance. Such investigations can inform the scientific renovation of residential spaces and the improvement of environmental support systems, ultimately enhancing older adults’ physical and mental well-being in the context of accelerating population aging.

As an important factor influencing residents’ health, the built environment encompasses both objective physical elements, such as green space systems, transportation networks, and land use structures, as well as residents’ subjective perceptions of and experiences with the environment. Existing research has demonstrated that environmental conditions contribute to socioeconomic health inequality, with exposure to adverse environmental factors disproportionately affecting the health of economically disadvantaged populations^8^. An optimized built environment can create living conditions conducive to public health, thereby effectively enhancing residents’ physical and mental health^9–11^. In this study, built environment is conceptualized along two distinct but interrelated dimensions. Objective built environment (OBE) refers to the tangible, measurable physical spaces constructed or modified by humans for dwelling, working, and leisure purposes. Drawing on the 5D conceptual framework proposed by Cervero et al.^12^ and contextualized to the distinctive spatial morphology of traditional Dong ethnic settlements, OBE is operationalized in this study through five dimensions: street connectivity, functional diversity, transportation accessibility, public space density, and service facility accessibility. Perceived built environment (PBE), by contrast, refers to older adults’ subjective experiences and cognitive evaluations of their residential environment, operationalized through five dimensions: transportation convenience, environmental safety, recreational and entertainment facilities, overall environmental aesthetics, and sense of community belonging. These dimensions reflect how individuals interpret and respond to objective environmental stimuli, were assessed using a 5-point Likert scale. Prior studies have demonstrated that objective environmental characteristics, such as higher street density, can reduce motor vehicle use and promote healthier travel behaviors including walking and cycling^13^. The provision of recreational, sports, and landscape facilities has also been shown to contribute positively to residents’ psychological health^14^. Nevertheless, examining residents’ health solely from the perspective of OBE remains insufficient. Such an approach fails to capture individuals’ subjective environmental experiences and psychological responses. Indeed, research has shown that residents’ satisfaction with their residential environment exerts a stronger influence on psychological states and behavioral intentions than objective environmental attributes, thereby generating more substantial health benefits^5^. Consequently, an increasing number of studies have attempted to integrate OBE with PBE to examine their interactive effects, recognizing that the pathway from physical space to health outcomes is often mediated by residents’ perceptual and behavioral responses.

Previous studies have examined the influence of built environment factors, including leisure facility availability, food store accessibility, and proximity to public transportation, on older adults’ leisure activities (LA) and HS. However, existing research has predominantly focused on urban communities. In contrast, limited attention has been paid to rural areas, particularly to traditional settlements of ethnic minorities in remote regions. These traditional settlements typically possess distinctive spatial configurations, lifestyles, and social organizational structures that differ significantly from urban communities in terms of environmental perception, behavioral preferences, and health risk profiles^15^. Consequently, expanding the scope of research to encompass rural traditional settlements is necessary, with particular attention to the distinctive pathways through which the built environment influences local residents’ health. Compared with urban communities, older adults in rural traditional settlements typically maintain more intimate neighbor relationships and daily interaction patterns; their LA are often not isolated physical behaviors but rather life practices deeply intertwined with social interaction^16^, exemplified by casual visiting and conversation, mutual aid among neighbors, collective labor, and courtyard gatherings. This integrated lifestyle pattern suggests that spatial configuration, activity convenience, and social accessibility may have more pronounced effects on older adults’ physical and mental health than is observed in urban settings. Therefore, it is imperative to incorporate LA and social participation (SP) as key behavioral mechanisms into the analytical framework to systematically examine their roles in the pathways through which built environment influences health.

Based on these considerations, this study selected traditional Dong ethnic settlements in Western Hunan as a research case to explore the mechanisms through which built environment influences older adults’ health, thereby providing theoretical reference and practical evidence for the optimization of rural built environments. This study attempts to address five interrelated research questions:

- RQ1: How do OBE and PBE respectively influence older adults’ HS, and what is the relationship between these two environmental dimensions?
- RQ2: Do LA and SP directly promote older adults’ health?
- RQ3: Is there an interaction between LA and SP, specifically, does LA facilitate SP?
- RQ4: Do LA and SP function as independent parallel mediators in the relationship between built environment and older adults’ health?
- RQ5: Beyond parallel mediation, do LA and SP form a sequential mediation pathway (LA → SP → HS) through which built environment influences older adults’ health?

To address these research questions, this paper systematically integrates theoretical discussion with empirical analysis. The paper is structured as follows: the introduction presents the research background and significance; the literature review constructs the research model and formulates hypotheses; the methods section describes the study area and data collection; the results section presents empirical findings and hypothesis validation; the discussion addresses implications; and the conclusion summarizes key findings and proposes directions for future research.

### Literature review and research hypotheses

A growing body of research has examined how the community built environment shapes older adults’ physical activity, social interaction, and psychological health. Across this literature, a recurring pattern emerges: environmental features such as green space accessibility, walkable streets, and mixed land use are consistently associated with health-promoting behaviors, yet the magnitude and direction of these associations vary considerably across contexts. Rather than reflecting methodological inconsistency, this variability points to a substantive theoretical insight: the relationship between the built environment and health is neither linear nor universal, but is mediated by residents’ perceptual responses and behavioral practices, and moderated by the social and cultural contexts in which environments are embedded. Translating objective spatial attributes into health outcomes thus requires analytical frameworks that capture both the perceptual and behavioral intermediaries through which environmental effects are realized. To this end, integrating OBE, PBE, LA, SP, and HS within a unified analytical chain offers a more theoretically complete approach to understanding these relationships.

Furthermore, existing research frameworks have been predominantly constructed within modern urban contexts, with theoretical assumptions rooted in individualized residential patterns emphasizing personal choice, private space, and independent daily behavioral patterns. In contrast, traditional settlement communities exhibit distinctive characteristics in both spatial morphology and social structure, typically demonstrating stronger collectivity and space-sharing qualities grounded in acquaintance networks. Their built environment not only serves residential and transportation functions but also provides key venues for daily LA and social interaction^17^, suggesting that built environment may exert effects on older adult health through indirect pathways that differ substantially from those identified in modern urban contexts.

Traditional Dong ethnic settlements in Western Hunan provide a distinctive context for examining relationships between the built environment and health. These settlements retain well-preserved spatial forms and sociocultural structures, allowing analysis within a non-urbanized, tradition-based setting largely unaffected by modern planning^18^. Organized around communal cores such as drum towers, ancestral halls, and covered bridges, they exhibit centripetal layouts that differ fundamentally from urban spatial configurations^19^. As a clan-based agrarian society, the Dong people maintain strong kinship ties, frequent intergenerational interaction^20^, and rich collective activities, fostering a pronounced sense of collective consciousness^21^. This socio-spatial context contrasts with the individual-oriented assumptions of most existing research and enables examination of how embedded social networks and communal space shape the pathways linking built environment and older adult health. At the same time, pronounced population aging and youth outmigration heighten the practical relevance of understanding environmental determinants of older adults’ health in these communities.

In recent years, research has shown that the effects of the built environment on older adult health are often mediated by behavioral and social mechanisms, particularly LA and SP. Studies indicate that supportive environments, such as accessible green spaces and walkable streets, promote LA and improve physical and cognitive health^22^. Similarly, built environment qualities including accessibility, safety, and aesthetics can indirectly enhance health by encouraging outdoor activities^23^. Regarding SP, pedestrian-friendly design and convenient transportation increase social interaction opportunities, strengthen social capital, and improve psychological well-being^24^. Moreover, higher-quality public spaces are associated with more frequent social activities and reduced loneliness and depression among older adults^25^.

**Fig. 1:**
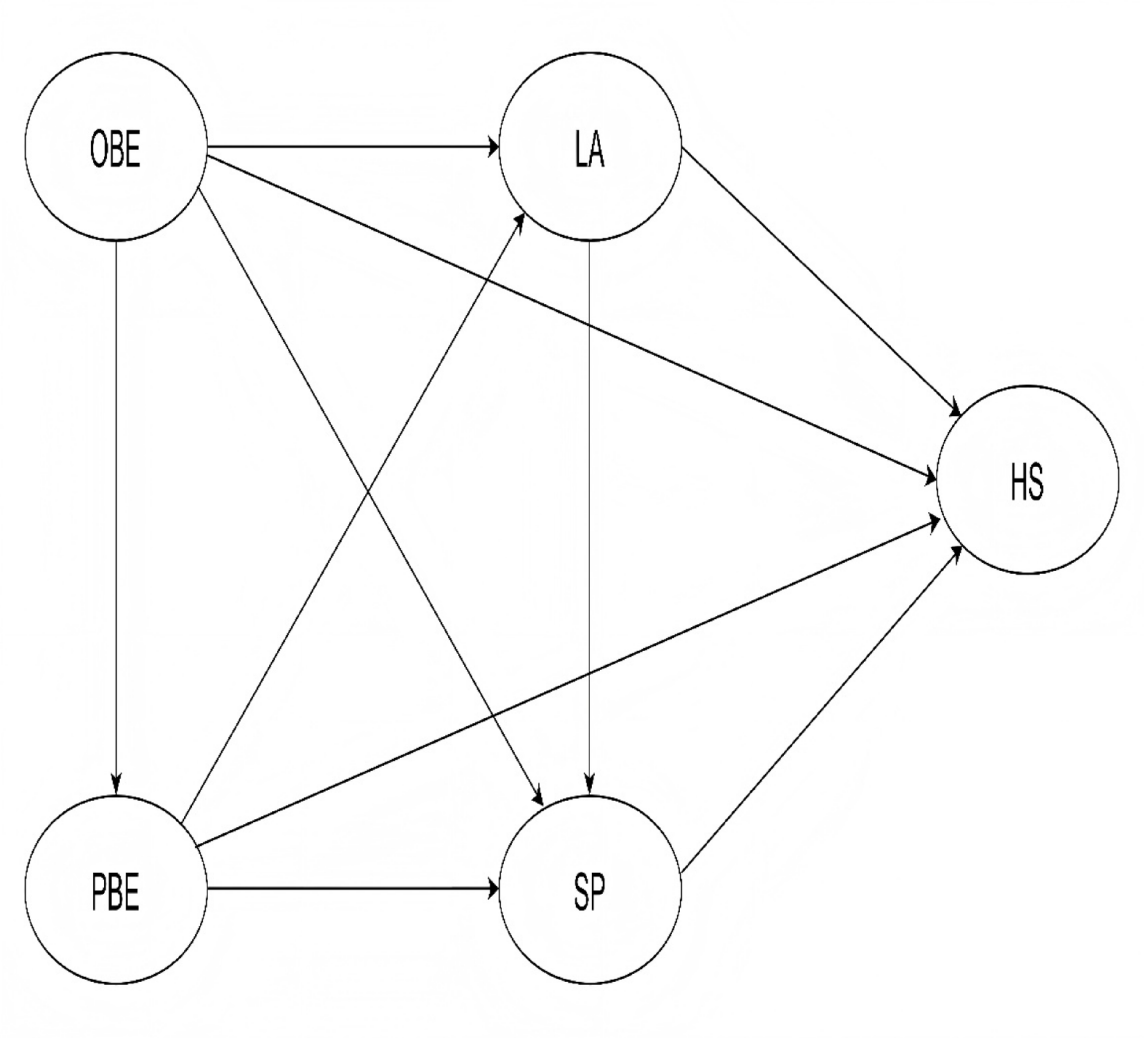
Conceptual framework: a combined parallel-and-serial mediation model.

A gap remains, however, in frameworks that treat LA and SP as simultaneously operating mechanisms. Most existing models examine these pathways in isolation, leaving open the question of whether LA and SP function not only as independent parallel mediators but also as sequential intermediaries in which leisure engagement shapes subsequent social participation. This study addresses this gap by constructing a combined parallel-and-serial mediation framework (Fig. 1) positing three distinct mediation pathways: parallel mediation through LA independently, parallel mediation through SP independently, and serial mediation whereby LA sequentially influences SP, which then affects health outcomes.

Within the aforementioned theoretical framework, existing research has demonstrated that OBE can directly generate health benefits. Numerous epidemiological studies have confirmed that improved air quality reduces risk of chronic diseases^26^, while increased neighborhood green space proportion alleviates psychological stress and positively affects disease management for conditions such as dementia and diabetes^27^. Furthermore, the effects of OBE on individual health also operate through the mediation of residents’ subjective perception: objective characteristics such as street greenery, quality of public facilities, and transportation accessibility are typically transformed into subjective evaluations of environmental safety, comfort, and convenience, which subsequently influence health-related behaviors and final health outcomes. Research evidence indicates that positive environmental perception stimulates increased physical activity, enhances psychological sense of safety and life satisfaction, and ultimately improves physical and mental health^28^. Based on these findings, this study proposes:

#### H1:

OBE is positively associated with PBE.

#### H2:

 PBE is positively associated with older adult HS.

#### H3:

OBE is positively associated with older adult HS.

The health-promoting effects of LA among older adults are well established across physiological, psychological, and cognitive domains, encompassing improvements in cardiovascular function, muscle strength, and bone density, as well as reductions in depression, anxiety, and cognitive decline^29,30^. Theoretically, these benefits are understood through both biological pathways, whereby physical movement directly conditions bodily systems, and psychosocial pathways, whereby engagement in meaningful activity enhances emotional regulation and self-efficacy. In traditional Dong ethnic settlements, older adults’ LA are characterized by strong spatial embeddedness and collective participation^31^: activities such as casual conversation at drum tower squares, resting at covered bridges, and walking along village roads are spatially anchored in communal public spaces. This spatial embeddedness suggests that the health-promoting capacity of LA in these settings is inseparable from the built environment that accommodates and structures such activity. Based on these findings, this study proposes:

#### H4:

 LA is positively associated with older adult HS.

SP constitutes a well-documented protective factor for older adult health, with evidence linking active social engagement to reduced psychological distress, lower risks of depression and cognitive decline, and enhanced subjective well-being^32,33^. These benefits are theoretically grounded in social capital theory, which holds that participation in collective networks generates trust, reciprocity, and informational resources that buffer against health-damaging stressors. In the context of traditional Dong settlements, where public spaces such as drum towers and covered bridges simultaneously serve as venues for daily LA and collective social practices, the boundary between leisure and social engagement is structurally blurred. This spatial co-location suggests that LA may function not only as an independent health-promoting behavior but also as a gateway through which older adults enter and sustain broader networks of social participation, thereby producing compounding health benefits through sequential behavioral pathways. Based on these considerations, this study proposes:

#### H5:

SP is positively associated with older adult HS.

#### H6:

 LA is positively associated with SP.

Both OBE and PBE may show indirect associations with older adult health through multiple mediation pathways. On one hand, favorable physical environments provide convenient and safe activity spaces for older adults, stimulating LA participation and improving cardiovascular function, emotional state, and overall health^34^. When older adults perceive community environments as safe and convenient, they are more inclined to engage in outdoor activities, thereby obtaining health benefits^35^. On the other hand, environmental elements such as mixed land use, pedestrian-oriented street design, and high-quality public green space create increased opportunities for social interaction, promoting neighbor interaction and formation of social support networks^6^. Similarly, positive subjective environmental perception enhances older adults’ sense of belonging and safety, thereby promoting SP behaviors^36^. Based on these findings, this study proposes:

#### H7:

LA independently mediates the relationship between OBE and older adult HS.

#### H8:

 LA independently mediates the relationship between PBE and older adult HS.

#### H9:

 SP independently mediates the relationship between OBE and older adult HS.

#### H10:

 SP independently mediates the relationship between PBE and older adult HS.

Beyond these parallel pathways, LA and SP may also form a serial mediation chain whereby built environment influences health sequentially through LA and then SP. Existing research indicates that collective LA such as square dancing, card games, and group walking not only directly promote physical and mental health but also strengthen interpersonal interaction and community cohesion, thereby expanding social networks^37^. Based on these considerations, this study proposes:

#### H11:

 LA and SP together function as sequential mediators in the relationship between OBE and older adult HS.

#### H12:

LA and SP together function as sequential mediators in the relationship between PBE and older adult HS.

To ensure transparency in the research design and facilitate reader comprehension, Table 1 systematically maps the five research questions (RQ1–RQ5) to the twelve hypotheses (H1–H12) and their corresponding structural model pathways.

**Table 1:** Mapping of research questions to hypotheses and structural model paths.

Research question	Hypothesis	Model path/relationship
RQ1: How do objective and perceived built environments influence older adults’ health, and what is the relationship between them?	H1, H2, H3	OBE → PBE; PBE → HS; OBE → HS
RQ2: Do leisure activities and social participation directly promote older adults’ health?	H4, H5	LA → HS; SP → HS
RQ3: Is there an interaction between leisure activities and social participation?	H6	LA → SP
RQ4: Do leisure activities and social participation function as independent parallel mediators in the relationship between built environment and older adults’ health?	H7, H8, H9, H10	OBE/PBE → LA → HS; OBE/PBE → SP → HS
RQ5: Do leisure activities and social participation form a sequential mediation pathway in the relationship between built environment and older adults’ health?	H11, H12	OBE/PBE → LA → SP → HS

### Research methods

#### Study area overview

This study focuses on Dong ethnic settlement clusters in Huaihua, Hunan Province, China, located in the southwestern part of Hunan Province (109°25′ to 110°E; 25°52′ to 26°29′N), covering a total area of 2,239 square kilometers. The region is surrounded by the Wuling and Xuefeng mountain ranges, with the Liuxi and Quxi river basins constituting the primary distribution areas of traditional Dong ethnic settlements. The distinctive mountainous terrain and relatively isolated geographic environment have enabled exceptional preservation of traditional Dong culture, spatial forms, and clan structures^38^. Dong ethnic settlements are primarily distributed across counties including Tongdao, Xinhuang, Zhijiang, Jingzhou, and Huitong, and are characterized by linear or clustered spatial configurations centered on traditional public structures—drum towers, meditation halls, pavilions, and covered bridges—which function not merely as physical entities but as primary carriers of social activity and cultural identity.

The relative geographic isolation of Dong settlements has preserved traditional landscapes and acquaintance-based social structures, creating conditions suitable for examining built environment effects beyond urban paradigms. From a practical standpoint, the region faces severe and compounding aging challenges: according to 2024 statistical data from Huaihua, the city’s permanent resident population is 4.4159 million, of which 927,300 (21.0%) are aged 60 and above. In the three primary Dong autonomous counties, aging rates are even more pronounced. Xinhuang Dong Autonomous County records 22.22% of the population aged 60 and above, Zhijiang Dong Autonomous County 23.12%, and Tongdao Dong Autonomous County 19.87%^39^, all exceeding the threshold of an aged society. These conditions are further exacerbated by large-scale youth outmigration to urban areas, rendering the older adult population increasingly reliant on the local built environment and community networks for health support. Additionally, compared to urban areas, rural regions exhibit relative deficiencies in socioeconomic characteristics, infrastructure development, and community service accessibility^40^. Investigating the mechanisms through which built environment influences older adult health in this context not only fills an important gap in research on traditional settlement environments but also provides empirical evidence for healthy aging policy in ethnic minority rural areas. Given that clan-based social organization, communally oriented spatial configurations, and aging-in-place trends driven by youth outmigration are defining features shared by many rural and traditional communities across East Asia, Southeast Asia, and beyond^41,42^, the mechanisms identified in this study may offer transferable insights well beyond the immediate Dong settlement context.

### Data sources

**Fig. 2:**
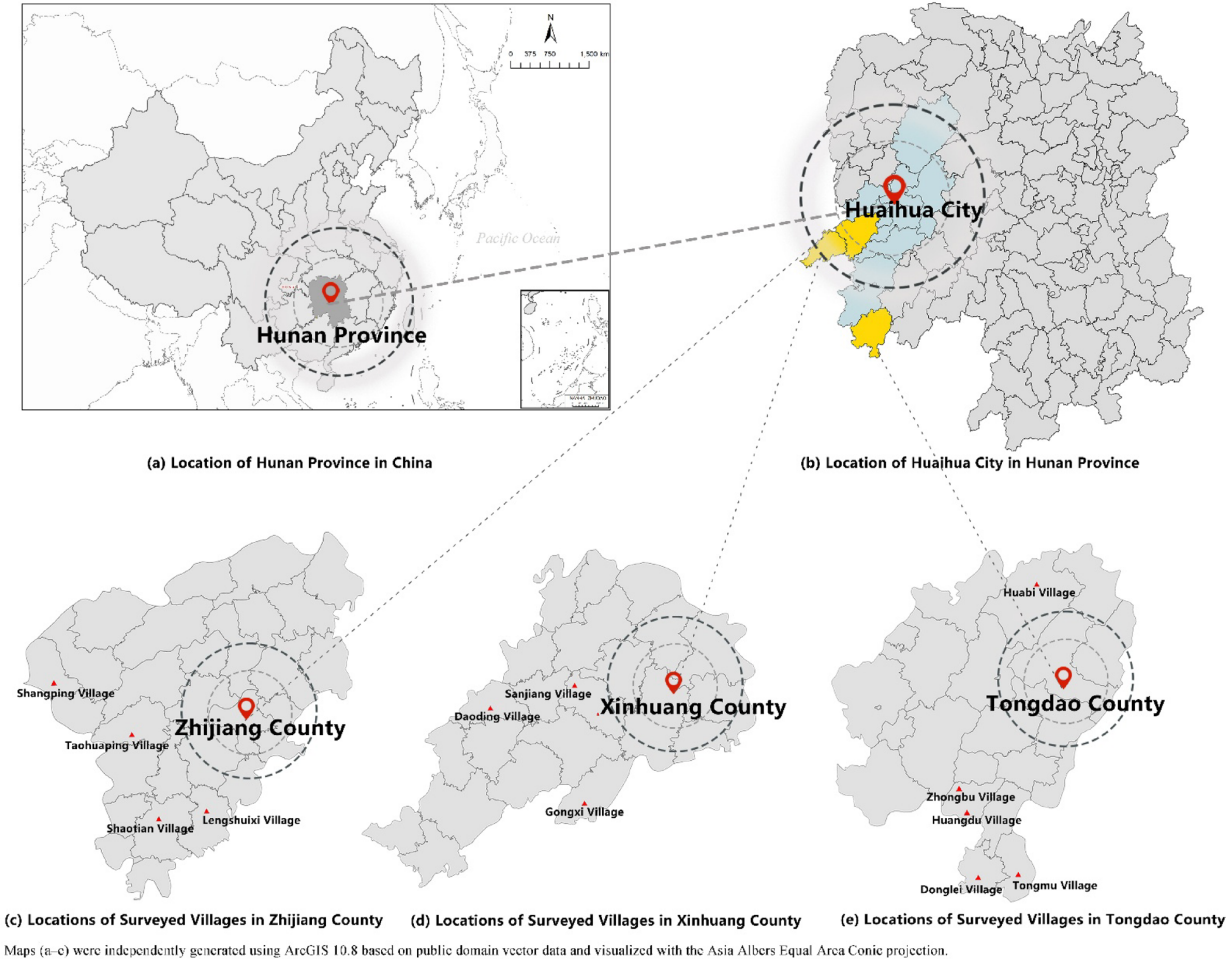
Distribution of the study area.

This study is based on traditional Dong ethnic settlements in three Dong autonomous counties in Huaihua, Hunan Province: Xinhuang, Tongdao, and Zhijiang counties. To ensure the representativeness of the sample and the rationality of regional distribution, the research team conducted an offline household survey in 12 randomly selected Dong villages (Gongxi, Daodin, Sanjiang, Tongmu, Taohuaping, Huangdu, Huabi, Zhongbu, Donglei, Lengshuixi, Shaotian, and Shangping villages) from May 25 to August 23, 2025, (see Fig. 2). With institutional and local village committee approval, surveys were conducted with guidance from local residents. Given that some respondents had limited educational backgrounds and dialect variations existed, surveyors employed oral explanation methods to clarify questionnaire content, ensuring consistent comprehension across respondents. This research protocol received approval from the Research Ethics Committee of Hunan University of Information Technology, and all participants provided written informed consent. No minors were involved in the investigation. All methods were performed in accordance with the relevant guidelines and regulations. To enhance participation motivation and data quality, the research team provided each surveyed household with a small cash incentive of 10 yuan as a token of appreciation. Following respondent completion of questionnaires, surveyors employed mobile terminals to input data in real-time on the “Questionnaire Star” platform while simultaneously recording supplementary information. To ensure data quality, the research team conducted item-by-item verification of all questionnaires, applying the following criteria to identify invalid responses: excessive missing data, excessively uniform response patterns, or failure to pass attention-check questions. A total of 216 questionnaires were distributed, yielding 187 valid responses with a valid response rate of 86.57%, meeting sample size requirements. Following data cleaning and organization, a final analytical dataset was established.

### Variable operationalization and measurement

#### Health status, leisure activities and social participation

This study measured older adult health status using a self-rated health scale encompassing physical and psychological dimensions. The physical health dimension assessed how physical condition affects daily activities and satisfaction with physical status, while the psychological dimension measured feelings of anxiety/depression and overall life satisfaction. Two mediating variables were also measured: LA frequency and SP intensity. Drawing upon established measurement frameworks^43–45^, LA frequency was assessed through four observed variables that were specifically adapted to reflect distinctive activity patterns in Dong ethnic settlements: (1) sports and fitness (including traditional group exercises such as collective morning tai chi in village squares); (2) entertainment activities (emphasizing communal forms such as antiphonal singing sessions and traditional Dong musical performances); (3) excursions and market activities (incorporating traditional “ganyue” market gatherings specific to ethnic minority regions); and (4) ethnic cultural activities (including participation in traditional festivals, drum tower gatherings, and wind-and-rain bridge social events—spatial-cultural practices unique to Dong settlements). Following multidimensional conceptualizations of social participation^46–48^, SP intensity was measured via four variables tailored to the “acquaintance society” structure of traditional Dong communities: (1) neighbor mutual aid (reflecting the “mutual assistance collective” tradition deeply embedded in Dong culture); (2) settlement governance (including participation in traditional clan council meetings and village assembly discussions held at drum towers); (3) clan activities (encompassing lineage-based ceremonies, ancestral worship, and kinship network maintenance—central to Dong social organization); and (4) public welfare services (including collective maintenance of communal infrastructure such as wind-and-rain bridges and participation in traditional “collective labor” practices). These measurement dimensions were selected because they represent institutionalized social practices that distinguish traditional Dong settlements from mainstream urban or Han Chinese rural communities, where individualized leisure and formal organizational participation tend to predominate.

All questionnaire-based variables were scored on 5-point Likert scales, with higher scores indicating better health status or greater participation levels. Measurement instruments were locally adapted through a three-stage process: (1) initial item pool development based on literature review and preliminary ethnographic observations; (2) expert consultation with local cultural specialists and community leaders to ensure items captured culturally salient practices; (3) cognitive interviews with older Dong residents (*n* = 15) to verify item comprehensibility and cultural relevance. The reliability and validity of the localized instruments were verified through a pilot study (*n* = 30) conducted prior to the formal survey. For the final analytical dataset, Cronbach’s α coefficients for all latent variables ranged from 0.75 to 0.88, indicating robust internal consistency.

### Objective built environment

OBE measurements were developed by reference to the 5D conceptual framework proposed by Cervero et al.^12^, combined with the spatial characteristics of traditional Dong ethnic settlements in Western Hunan and daily activity patterns of older adults. While the five OBE dimensions (street connectivity, functional diversity, transportation accessibility, public space density, and service facility accessibility) follow conventional urban planning metrics, their operationalization was contextualized to the distinctive spatial morphology of Dong settlements: (1) “street nodes” include not only conventional road intersections but also traditional pathways connecting residential clusters, drum towers, and wind-and-rain bridges; (2) “public spaces” were defined to encompass culturally specific communal venues such as drum tower squares, covered bridges, and clan ancestral halls—spaces that serve as the social heart of Dong villages and differ fundamentally from generic urban plazas; (3) “functional spaces” included handicraft workshops (e.g., traditional textile and silverwork studios) and ethnic cultural facilities unique to Dong settlements. This contextualization ensures that OBE measurements capture the built environment features that are functionally and culturally meaningful to older Dong residents, rather than imposing urban-centric definitions onto a traditional rural setting. Existing research demonstrates that older adults’ activity ranges are relatively limited; compared to younger populations, smaller buffer zones are more appropriate for environmental characteristic assessment^49^. Ding’s research further confirmed that built environment characteristics within 500 m of residence demonstrated stronger explanatory power for predicting older adult physical activity compared to characteristics within 1 to 1.6 km ranges^50^. Based on the aforementioned evidence, this study employed a 500-meter buffer zone for spatial analysis.

**Fig. 3:**
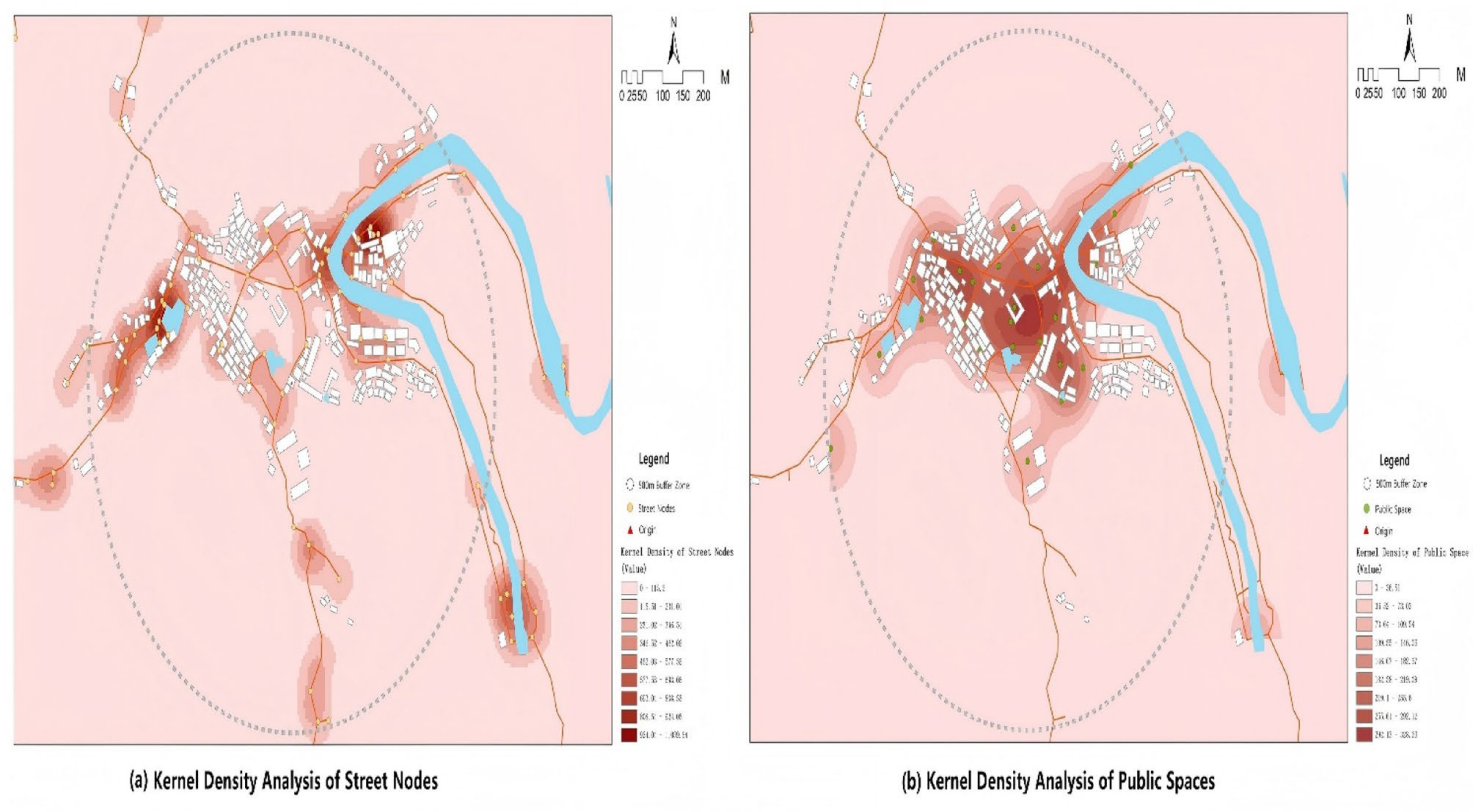
Kernel density analysis of Huangdu Village within a 500 m radius. Maps were generated by the authors in ArcGIS 10.8 (Esri Inc., Redlands, CA, USA; https://www.esri.com).

**Fig. 4:**
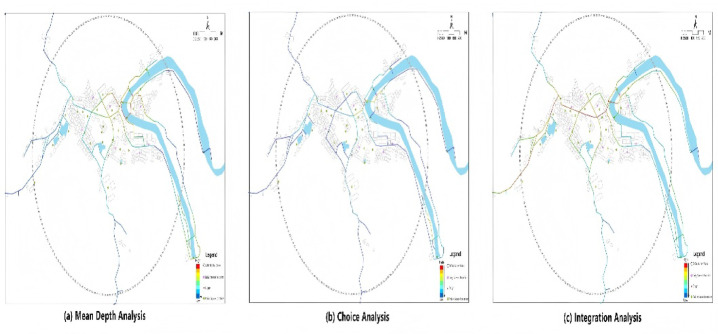
Street network structure analysis of Huangdu Village within a 500 m radius. Spatial syntax computations were performed using depthmapX (version 0.8.0; https://github.com/SpaceGroupUCL/depthmapX).

**Fig. 5:**
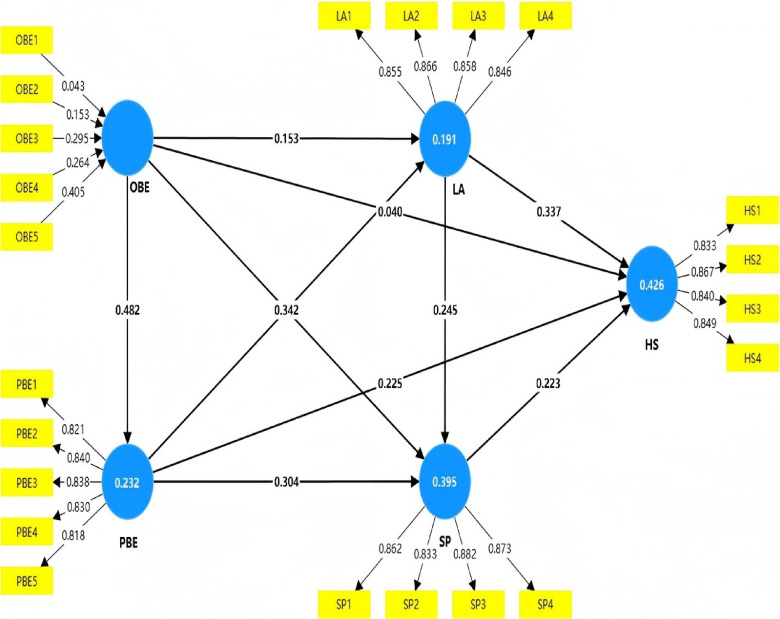
PLS-SEM results of built environment factors. influencing older adults’ health in traditional dong settlements.

The specific measurement indicators are as follows: (1) Street node density: calculated the density of street intersection nodes within the buffer zone, reflecting the spatial connectivity of traditional settlements (see Fig. 3a); (2) Functional space diversity: calculated the proportional distribution of different functional spaces (such as residential buildings, commercial facilities, handicraft workshops, and public buildings) within the buffer zone using entropy indices, reflecting the mixed-use degree and spatial diversity of settlement functions; (3) External transportation accessibility: measured by walking network distance to the nearest major road, this indicator directly reflects the convenience of daily travel and external connections for settlement residents The underlying street network structure, characterized through spatial syntax analysis, is illustrated in Fig. 4. (4) Public space location density: calculated the density of public space locations within the buffer zone available for residents’ daily leisure, neighbor interaction, and cultural activities (see Fig. 3b); (5) Daily service facility accessibility: calculated walking network distance from residence to the nearest essential service facilities (such as village health clinics and shops), reflecting the convenience for settlement residents to access daily living services. All objective spatial data were obtained through GPS field measurement, high-resolution satellite image interpretation, and field investigation. To ensure consistent interpretation of all OBE indicators, distance-based accessibility variables (external transportation accessibility and daily service facility accessibility) were reverse-coded prior to z-score normalization, such that higher values represent better accessibility (shorter distances). The transformation was performed using: Accessibility = max (Distance) - Distance_original. This coding procedure ensures that positive path coefficients in the structural equation model consistently represent health-promoting environmental characteristics across all OBE dimensions. Finally, all indicators underwent z-score normalization ($\:Z=(x-\mu\:)/\sigma\:$) to transform them into uniform dimensions, facilitating subsequent statistical analysis. The specific measurement indicators and their mathematical constructions are standardized in Table 2.

**Table 2:** Operational definitions and calculation formulas for OBE indicators.

Indicator	Formula / definition	Unit	Operational rules and parameter specifications
Street node density ($\:{\mathrm{D}}_{\mathrm{n}}$)	$\:{\mathrm{D}}_{\mathrm{n}}\mathrm{=}\frac{\mathrm{N}}{\mathrm{A}}$	$\:\mathrm{nodes}\mathrm{/}\mathrm{k}{\mathrm{m}}^{\mathrm{2}}$	$\:\mathrm{N}$: Number of intersections with 3 or more road segments within the buffer; $\:\mathrm{A}$: Buffer area ($\:\mathrm{0.785}\mathrm{k}{\mathrm{m}}^{\mathrm{2}}$).
Functional space diversity ($\:\mathrm{H}$)	${\mathrm{H}}\,{\text{ = - }}\,\frac{{\sum {_{{i = 1}}^{n} \left( {P_{i} \, \times \ln P_{i} } \right)} }}{{{\mathrm{lnn}}}}$	Dimensionless	Shannon Entropy Index. $\:{\mathrm{P}}_{\mathrm{i}}$: Area proportion of the $\:{\mathrm{i}}^{\mathrm{t}\mathrm{h}}$ land-use type; $\:\mathrm{n}$: Total categories (Residential, Commercial, Handicraft, Public, Service).
External transportation accessibility ($\:{\mathrm{A}}_{\mathrm{t}}$)	$\:{\mathrm{A}}_{\mathrm{t}}\mathrm{=}\mathrm{min}\mathrm{(}\mathrm{Dis}{\mathrm{t}}_{\mathrm{road}}\mathrm{)}$	$\:\mathrm{m}$	Shortest walking network distance from the household to the nearest major external road.
Public space location density ($\:{\mathrm{D}}_{\mathrm{p}}$)	$\:{\mathrm{D}}_{{\mathrm{p}}} {\text{ = }}\frac{{{\mathrm{S}}_{{\mathrm{p}}} }}{{\mathrm{A}}} \times {\mathrm{100}}\%$	$\:\mathrm{\%}$	$\:{\mathrm{S}}_{\mathrm{p}}$: Total area of public leisure spaces (e.g., Drum Tower squares, opera stages, communal wells) within the buffer.
Daily service facility accessibility ($\:{\mathrm{A}}_{\mathrm{s}}$)	$\:{\mathrm{A}}_{\mathrm{s}}\mathrm{=}\mathrm{min}\mathrm{(}\mathrm{Dis}{\mathrm{t}}_{\mathrm{fac}}\mathrm{)}$	$\:\mathrm{m}$	Shortest walking network distance from the household to the nearest clinic or village shop.

### Perceived built environment

PBE was assessed via a structured questionnaire designed to capture older adults’ subjective evaluations and cognitive experiences of their residential environment. Five observed variables were employed for measurement: transportation convenience in residential areas, environmental safety conditions, recreational and entertainment facilities, overall environmental aesthetics, and sense of community belonging. The recall windows were tailored to the nature of the activities: a one-week window was used for high-frequency LA to minimize recall bias, while a one-year window was applied to episodic SP items (e.g., clan activities, village governance) to capture participation in seasonal or rare events that would otherwise be missed in shorter timeframes. Scores were assessed using a 5-point Likert scale (1 = very dissatisfied, 5 = very satisfied), with higher scores indicating more positive older adult perceived evaluation of the environment. Specific measurement items for each observed variable are presented in Table 3.

**Table 3:** Questionnaire items and variable descriptions.

Latent variables	Observed variables	Calculating methods
Objective built environment	Street intersection density	Density of street intersections within a 500 m buffer.
	Land use mix	Proportional distribution of different land use types within a 500 m buffer.
	Accessibility to major roads	Pedestrian network distance from the residence to the nearest major road within a 500 m buffer.
	Density of Public Spaces	Density of public spaces available for residents’ daily leisure, social interaction, and cultural activities within a 500 m buffer.
	Accessibility to Essential Amenities	Pedestrian network distance from the residence to the nearest essential service facilities (e.g., village clinic, local store).
Perceived environment	How satisfied are you with the convenience of transportation in your current residential area?	1 (very dissatisfied) ~ 5 (very satisfied)
	How satisfied are you with the environmental safety and security in your current residential area?	1 (very dissatisfied) ~ 5 (very satisfied)
	How satisfied are you with the recreational and leisure facilities in your current residential area?	1 (very dissatisfied) ~ 5 (very satisfied)
	How satisfied are you with the overall aesthetic appeal of your current residential area?	1 (very dissatisfied) ~ 5 (very satisfied)
	How satisfied are you with your sense of belonging in your current residential area?	1 (very dissatisfied) ~ 5 (very satisfied)
Leisure activities	In the past week, how often did you participate in physical exercises (e.g., walking, Tai Chi, morning workouts, jogging)?	1 (0 times) ~ 5 (every day)
	In the past week, how often did you participate in cultural and recreational activities (e.g., watching outdoor movies, playing chess/cards, singing, calligraphy, painting, handicrafts)?	1 (0 times) ~ 5 (every day)
	In the past week, how often did you participate in outings (e.g., visiting a country market, attending a temple fair, rural sightseeing)?	1 (0 times) ~ 5 (every day)
	In the past week, how often did you participate in ethnic cultural activities (e.g., Dong Grand Song, Dong opera, festivals, ethnic dances)?	1 (0 times) ~ 5 (every day)
Social participation	In the past year, how often did you participate in neighborhood mutual help activities (e.g., helping neighbors with farm work, babysitting, lending/borrowing tools, visiting sick neighbors)?	1 (0 times) ~ 5 (10 or more times)
	In the past year, how often did you participate in village public affairs (e.g., attending village assemblies, contributing to public facility construction, participating in village governance decisions)?	1 (0 times) ~ 5 (10 or more times)
	In the past year, how often did you participate in clan activities (e.g., attending ancestral worship ceremonies, clan gatherings, renovating ancestral halls)?	1 (0 times) ~ 5 (10 or more times)
	In the past year, how often did you participate in voluntary or charitable activities (e.g., environmental clean-ups, poverty alleviation, disaster relief donations)?	1 (0 times) ~ 5 (10 or more times)
Health status	To what extent does your current physical condition limit you in performing daily activities (e.g., housework, shopping, bathing, dressing)?	1 (severely limited) ~ 5 (not limited at all)
	Overall, how satisfied are you with your current physical health condition?	1 (very dissatisfied) ~ 5 (very satisfied)
	In the past month, how often have you experienced negative emotions (e.g., anxiety, worry, depression, feeling down)?	1 (always) ~ 5 (never)
	Overall, how satisfied are you with your current life as a whole (including family relations, economic status, living environment, and physical/mental health)?	1 (very dissatisfied) ~ 5 (very satisfied)

### Statistical analysis

In this study, SPSS 23.0 software was employed for data cleaning, preprocessing, and descriptive statistical analysis of sample demographic characteristics, while SmartPLS 4.0 software was utilized to construct a Partial Least Squares Structural Equation Modeling (PLS-SEM) framework to test the research hypotheses. The selection of the PLS-SEM path modeling approach was primarily based on the following considerations: first, PLS-SEM imposes relatively relaxed requirements on data distribution and does not necessitate strict normality of sample data, thereby demonstrating superior robustness when handling complex empirical data in social science research^51^. Second, the research model aimed to predict the effects of the built environment on older adults’ health and incorporated multiple mediating pathways such as leisure activities and social participation, and PLS-SEM exhibits significant advantages in handling such complex predictive models that are oriented toward theory development and maximization of variance explained in key target variables^52^. More critically, the latent variable of “OBE” in this study constitutes a formative construct defined by multiple indicators that are not necessarily highly correlated, and the PLS-SEM algorithm can effectively estimate path relationships involving formative measurement models, which traditional covariance-based structural equation modeling (CB-SEM) struggles to handle accurately. Therefore, this study adopted a two-stage analytical approach. In the first stage, the reliability and validity of the measurement model were assessed using indicators such as factor loadings, average variance extracted (AVE), and composite reliability (CR). In the second stage, the structural model was evaluated, with the significance of path coefficients tested through bootstrapping and the mediating effects examined in terms of their magnitude and significance. The results are presented sequentially in the subsections Evaluating the Measurement Model and Evaluating the Structural Model.

## Results

### Demographic characteristics of the sample

This study included 187 older adults individuals from traditional Dong ethnic settlements in Western Hunan as valid respondents, whose detailed demographic characteristics are presented in Table 4. The overall sample exhibited the following characteristics: in terms of gender composition, female respondents (*n* = 106, 56.7%) slightly outnumbered male respondents (*n* = 81, 43.3%). Regarding age structure, the sample was mainly composed of younger-old adults, with those aged 60–79 years accounting for 93.0% of the total (including 52.4% aged 60–69 years and 40.6% aged 70–79 years), while the oldest-old group aged 80 years and above represented a relatively small proportion (7.0%), which is consistent with the general difficulty of recruiting this cohort in field-based rural surveys, as reduced mobility and cognitive decline tend to limit participation; findings should therefore be interpreted with caution regarding generalizability to this subgroup. In terms of socioeconomic background, the educational attainment of the respondents was generally low, with nearly 80% (*n* = 148, 79.1%) holding primary school education or below, while only 7.4% had attained senior high school education or higher. The occupational composition was dominated by agricultural workers (*n* = 120, 64.2%), which is consistent with the rural characteristics of the study area. Nonetheless, educational level may moderate the associations between perceived built environment and health outcomes, a consideration relevant to cross-population generalization. In addition, personal monthly income levels were relatively low, with more than two thirds of the respondents (*n* = 125, 66.8%) earning less than 1,000 Chinese Yuan per month. Regarding living arrangements, the vast majority of older adults resided with family members (*n* = 155, 82.9%), while 17.1% (*n* = 32) lived alone. Moreover, the sample demonstrated strong residential stability, with 93.0% (*n* = 174) of respondents having resided locally for more than 20 years, indicating their long-term and deep integration into the local built environment, thereby providing a reliable sample foundation for investigating the cumulative health effects of the built environment. These sociodemographic characteristics are directly relevant to the research focus, as they collectively reflect the social structure, daily behavioral patterns, and living conditions typical of older adults in rural ethnic minority communities, providing essential context for interpreting the observed associations between built environment, behavioral mediators, and health outcomes.

**Table 4:** Demographic information of the participants.

Demographic factor	Categorie	Frequency	Percentage (%)
Gender	Male	81	43.3
	Female	106	56.7
Age	60–69 years	98	52.4
	70–79 years	76	40.6
	80–89 years	11	5.9
	90 years and above	2	1.1
Education level	Primary school or below	148	79.1
	Junior high school	25	13.4
	Senior high school	10	5.3
	College degree or above	4	2.1
Occupation	Agricultural worker	120	64.2
	Business/Service industry worker	36	19.3
	Migrant worker	24	12.8
	Village cadre/Community service staff	7	3.7
Personal monthly income	0-1000 RMB	125	66.8
	1001–2000 RMB	42	22.5
	2001–3000 RMB	11	5.9
	3001–4000 RMB	6	3.2
	Above 4000 RMB	3	1.6
Living arrangement	Living alone	32	17.1
	Living with family	155	82.9
Length of residence	10–20 years	13	7.0
	More than 20 years	174	93.0

### Evaluating the measurement model

Following the two-stage analytical approach, this study first assessed the measurement model. As shown in Table 5, all reflective latent variables (PBE, LA, SP, and HS) demonstrated satisfactory reliability and validity. All reflective constructs demonstrated satisfactory reliability and validity (Table 5). Internal consistency was confirmed across all constructs, with convergent validity supported by factor loadings exceeding 0.818 and AVE values ranging from 0.688 to 0.744. Discriminant validity was established via HTMT ratios, with a maximum value of 0.618, well below the 0.85 threshold (Table 6). Detailed indices are reported in Tables 5 and 6.

**Table 5:** Reliability and validity indicators of reflective latent variables.

Constructs	Items	Loadings	Cronbach’s α	CR	AVE
Perceived built environment	PBE1	0.821	0.887	0.888	0.688
	PBE2	0.840			
	PBE3	0.838			
	PBE4	0.830			
	PBE5	0.818			
Leisure activity	LA1	0.855	0.879	0.883	0.733
	LA2	0.866			
	LA3	0.858			
	LA4	0.846			
Social participation	SP1	0.862	0.886	0.890	0.744
	SP2	0.833			
	SP3	0.882			
	SP4	0.873			
Health status	HS1	0.833	0.869	0.874	0.718
	HS2	0.867			
	HS3	0.840			
	HS4	0.849			

**Table 6:** Discriminant validity (HTMT).

	HS	LA	PBE	SP
HS				
LA	0.618			
PBE	0.568	0.465		
SP	0.577	0.510	0.595	

Table 7 presents the evaluation results for the formative measurement model of OBE. Collinearity was assessed using variance inflation factors following recommended criteria for formative constructs^53^, with all VIF values (2.264–2.661) well below the threshold of 3.3, indicating acceptable levels. Outer weights analysis showed that OBE5 (daily service facility accessibility) attained statistical significance (0.405, *p* < 0.05), while OBE1–OBE4 weights were nonsignificant; nevertheless, all indicators demonstrated adequate empirical relevance, with outer loadings ranging from 0.804 to 0.901. The non-significant weights of OBE1 (street node density) and OBE2 (functional space diversity) are interpretable within the morphological characteristics of traditional Dong settlements, where built environment dimensions exhibit limited independent variation due to the organically integrated spatial structure of these villages. By contrast, OBE5 captures a dimension with more functionally differentiated relevance to older adults’ daily life, accounting for its singular significance. All five indicators were nonetheless retained on the grounds of content validity: each represents a conceptually distinct dimension of the built environment grounded in the 5D framework, and the removal of any indicator would compromise construct coverage. Consistent with formative measurement principles, content validity takes precedence over statistical significance in retention decisions.

**Table 7:** Measurement model evaluation for the formative construct OBE.

Construct	Items	Outer weight	T-statistics	*P*-values	Outer loading	VIF
Objective built environment	OBE1	0.043	0.210	0.834	0.804	2.661
	OBE2	0.153	0.797	0.426	0.822	2.503
	OBE3	0.295	1.752	0.080	0.847	2.264
	OBE4	0.264	1.484	0.138	0.854	2.449
	OBE5	0.405	2.177	0.029*	0.901	2.486

*p < 0.05.

### Evaluating the structural model

Following the assessment of the measurement model, this study examined the structural model using Cohen’s^54^ effect size criteria. The model exhibited satisfactory explanatory and predictive power overall, accounting for 42.6% of the variance in older adults’ HS (R² = 0.426), and all endogenous variables demonstrated predictive relevance (Q² > 0), indicating stable predictive capability of the model. The following results are organized in accordance with the hypothesized pathways in the research model, with direct associations examined first, followed by parallel and serial mediation effects.

In the analysis of direct associations, the OBE showed a robust link with the PBE (β = 0.482, 95% CI = [0.382, 0.590]) with a medium effect size approaching large (f² = 0.303); thus, H1 was supported. Regarding health outcomes, the PBE (β = 0.225, 95% CI = [0.098, 0.350]), LA (β = 0.337, 95% CI = [0.206, 0.456]), and SP (β = 0.223, 95% CI = [0.077, 0.362]) all demonstrated positive associations with older adults’ HS, supporting H2, H4, and H5. However, as all three paths exhibited small effect sizes (f² ranging from 0.052 to 0.149), they should be interpreted as modest contributing factors rather than primary determinants of health variance. Additionally, LA facilitated SP (β = 0.245, 95% CI = [0.112, 0.359]), supporting H6. Conversely, the direct association between OBE and HS was not significant (β = 0.040, 95% CI = [-0.095, 0.183]) with a negligible effect size (f² = 0.002), meaning H3 was not supported. Taken together, these direct path results indicate that OBE does not exert a direct influence on older adults’ health but operates through intermediate variables, whereas PBE exerts both direct and indirect effects.(see Tables 8 and 9).

**Table 8:** Explanatory power indicators of the structural model.

Dependent variables	*R*²	Q²	Path	f²	Effect size
HS	0.426	0.297	LA → HS	0.149	Small
			PBE → HS	0.055	Small
			OBE → HS	0.002	Negligible
			SP → HS	0.052	Small
LA	0.191	0.134	OBE → LA	0.022	Small
			PBE → LA	0.111	Small
PBE	0.232	0.155	OBE → PBE	0.303	Medium
SP	0.395	0.285	LA → SP	0.080	Small

Effect sizes classified following Cohen (1988): f² < 0.02 = negligible, 0.02 ≤ f² < 0.15 = small, 0.15 ≤ f² < 0.35 = medium, f² ≥ 0.35 = large. R² represents the coefficient of determination indicating the proportion of variance explained. Q² is Stone-Geisser’s Q² statistic assessing predictive accuracy, with values above zero indicating predictive capability.

**Table 9:** The coefficients for influence pathways of the structural model.

Hypothesis	Path relationship	Path coefficient (β)	T-value	*P*-value	95% CI (LL)	95% CI (UL)	Decision
H1	OBE → PBE	0.482	8.935	0.000***	0.382	0.590	Supported
H2	PBE → HS	0.225	3.498	0.000***	0.098	0.350	Supported
H3	OBE → HS	0.040	0.564	0.573	-0.095	0.183	Not supported
H4	LA → HS	0.337	5.341	0.000***	0.206	0.456	Supported
H5	SP → HS	0.223	3.031	0.002**	0.077	0.362	Supported
H6	LA → SP	0.245	3.890	0.000***	0.112	0.359	Supported

***p < 0.001, **p < 0.01, *p < 0.05. Significance determined using bootstrapping (5,000 resamples). Hypotheses supported when *p* < 0.05 and 95% CI excludes zero.

To elucidate the underlying mechanisms through which the built environment influences older adults’ health, both parallel and serial mediation effects of LA and SP were examined using bias-corrected bootstrapping (5,000 resamples) following Preacher and Hayes^55^. Full mediation was identified when the indirect effect was significant while the direct effect was not; partial mediation was identified when both effects were significant. Analyses addressed parallel mediation through LA and SP independently, as well as serial mediation through the LA→SP pathway. Results indicated that LA played a significant full mediating role between the OBE and HS (indirect effect = 0.052, 95% CI = [0.006, 0.117]); thus, H7 was supported. Similarly, SP also served as a significant full mediator between the OBE and HS (indirect effect = 0.057, 95% CI = [0.014, 0.119]), supporting H9. For the perceived built environment, the indirect effects of both LA (indirect effect = 0.115, 95% CI = [0.052, 0.182]) and SP (indirect effect = 0.068, 95% CI = [0.021, 0.125]) were significant, and given that the direct effect was also significant, this indicated that both served as partial mediators between the PBE and HS; therefore, H8 and H10 were supported. Collectively, these findings indicate that LA and SP constitute key behavioral pathways through which built environment characteristics are translated into older adults’ health outcomes. The full mediation of OBE effects through both LA and SP suggests that objective spatial attributes do not independently determine health; rather, their relevance is contingent upon residents’ active behavioral engagement. The partial mediation observed for PBE further underscores the compound role of subjective environmental perception, which operates on health both directly and through the facilitation of leisure and social behaviors.

Finally, this study examined the chain mediating effects. Analysis revealed that the chain mediation path “OBE → LA → SP → HS” was not significant (indirect effect = 0.008, 95% CI = [0.000, 0.024]); thus, H11 was not supported. In contrast, the chain mediation path “PBE → LA → SP → HS” demonstrated statistical significance (indirect effect = 0.019, 95% CI = [0.004, 0.037]), supporting H12 (see Table 10). Notably, the varying recall windows potentially complicate chronological mediation interpretation. This design is thus treated as a “representative steady state” of lifestyle patterns rather than a strict chronological sequence. The observed mediation effects reflect stable behavioral associations and the cumulative impact of these activity patterns on health status. In summary, the bootstrapping-based mediation analysis systematically revealed multiple pathways through which the built environment influences older adults’ health. The model results systematically revealed multiple pathways through which the built environment influences older adults’ health, wherein the effects of the OBE were fully mediated by LA and SP, while the PBE exerted compound influences on health through both direct paths, single mediation paths, and chain mediation paths simultaneously (see Fig. 5).

**Table 10:** Mediation effect results.

Hypothesis	Indirect path	Indirect effect	95% CI (LL)	95% CI (UL)	Mediation type	Decision
H7	OBE → LA → HS	0.052	0.006	0.117	Full	Supported
H8	PBE → LA → HS	0.115	0.052	0.182	Partial	Supported
H9	OBE → SP → HS	0.057	0.014	0.119	Full	Supported
H10	PBE → SP → HS	0.068	0.021	0.125	Partial	Supported
H11	OBE → LA → SP→ HS	0.008	0.000	0.024	None	Not Supported
H12	PBE → LA → SP→ HS	0.019	0.004	0.037	Partial	Supported

Significance assessed using bias-corrected bootstrapping (5,000 resamples). Mediation type: Full = indirect effect significant and direct effect not significant; Partial = both effects significant; None = indirect effect not significant.

### Common method bias assessment and Robustness check

Common method bias was addressed through both procedural and statistical measures. Procedurally, OBE was assessed via GIS-based spatial analysis independent of self-report, and questionnaire items were ordered to create psychological distance between predictor and outcome measures. Statistically, Harman’s single-factor test indicated that the first unrotated factor accounted for 44.5% of total variance, below the 50% threshold^56^, and full collinearity VIF values for all self-reported constructs ranged from 2.264 to 2.661, well within acceptable limits, suggesting that common method bias does not pose a substantial threat to the validity of the findings. To further verify the stability of the results, a robustness check was conducted by incorporating age, gender, education, monthly income, and living arrangement as control variables. As shown in Table 11, key path coefficients remained stable after covariate adjustment. None of the demographic control variables attained statistical significance as direct predictors of health status (all *p* > 0.05), and effect sizes across all covariates were negligible (f² < 0.02), confirming that built environment perceptions and behavioral practices constitute the primary predictors of health variance in this context.

**Table 11:** Results of the robustness check with sociodemographic covariates.

Hypothesis	Path	Without controls	With controls	Δβ	Significance
H1	OBE → PBE	0.482	0.482	0.000	Stable
H2	PBE → HS	0.225	0.231	+ 0.006	Stable
H3	OBE → HS	0.040	0.042	+ 0.002	Stable
H4	LA → HS	0.337	0.304	-0.033	Stable
H5	SP → HS	0.223	0.209	-0.014	Stable
H6	LA → SP	0.245	0.245	0.000	Stable
H7	OBE → LA → HS	0.052	0.047	-0.005	Stable
H8	PBE → LA → HS	0.115	0.104	-0.011	Stable
H9	OBE → SP → HS	0.057	0.053	-0.004	Stable
H10	PBE → SP → HS	0.068	0.064	-0.004	Stable
H12	PBE → LA → SP → HS	0.019	0.018	-0.001	Stable

Control variables include age, gender, education level, monthly income, and living arrangement. Maximum coefficient change = 0.033 (< 5% threshold).

## Discussion

### Relationship between the built environment and health

This study systematically investigated the pathways through which the built environment influences older adults’ health in traditional Dong ethnic settlements in Western Hunan. The findings revealed that OBE was significantly positively associated with PBE, which in turn showed direct positive associations with older adults’ HS, consistent with previous research suggesting that objective environmental characteristics are related to health through residents’ subjective experiences. For instance, Zhang et al.^57^ noted that OBE can significantly shape individuals’ perceptions and cognitions of their surrounding environment, thereby influencing their activity choices, while Yue et al.^49^ found that the diversity of leisure, landscape, and sports facilities within communities in Dalian was positively correlated with older adults’ mental health and could operate through the mediation of PBE. These associations have also been corroborated by similar studies in other regions^58,59^. While the OBE–PBE–health pathway is broadly consistent with findings from urban contexts, this study extends the evidence base by showing that the same perceptual mediation mechanism also operates in collectivist, tradition-based settlements, where spatial configurations, social structures, and daily behaviors differ markedly from those in mainstream built environment research. This finding strengthens the generalizability of the perceptual mediation framework beyond modern urban settings.

In the context of traditional Dong settlements, OBE characteristics such as high-density street networks, mixed-use land patterns, and abundant public interaction spaces tended to be perceived by older residents as a convenient, secure, and socially vibrant environment. The present findings substantiate this mechanism empirically: OBE demonstrated a robust association with PBE (β = 0.482, f² = 0.303), which in turn showed a significant direct association with health outcomes (β = 0.225)^60^. Importantly, this perceptual pathway carries a pronounced emotional dimension. Public spaces such as drum towers and wind-and-rain bridges function as emotionally charged arenas where older adults enact cultural rituals and reinforce collective identity; the affective responses elicited, including sense of security, belonging, and environmental satisfaction, appear to constitute the active mechanism through which spatial configuration becomes health-relevant, a process consistent with place attachment theory. Nevertheless, the small effect sizes associated with these paths (f² < 0.15) indicate that environmental perception functions as an incremental contributor rather than a primary determinant of health variance.

A more theoretically significant finding was that the direct association between OBE and health was not significant, diverging from urban studies in which accessibility to green spaces and public facilities has been linked directly to residents’ health^61,62^.

In urban contexts, neighborhood environmental attributes including street connectivity and walkability have been consistently linked to physical activity levels and weight status across multiple countries^13^, and the provision of recreational and landscape facilities has been associated with improved psychological health through direct exposure pathways^14^. These urban findings rest on an implicit assumption of environmental determinism: that objective spatial conditions translate directly into behavioral and health outcomes. The present findings challenge this assumption in the context of traditional rural settlements, where complete mediation via perceptual and social-behavioral pathways suggests that the mechanism linking built environment to health differs fundamentally from that observed in modern urban communities.

Two theoretical perspectives help account for this divergence. Drawing on Attention Restoration Theory^63^, environmental stimuli are unlikely to generate health benefits independently of residents’ cognitive and affective responses; rather, objective spatial features require subjective interpretation before their health-relevant effects are realized. In traditional Dong settlements, the health value of public spaces such as drum towers and wind-and-rain bridges resides not in their physical dimensions but in their function as sites of collective gathering, cultural ritual, and social recognition. The meanings residents ascribe to these spaces, reflected in this study through PBE, thus constitute the active mechanism through which OBE becomes health-relevant. This reasoning is further supported by Place Attachment Theory^64,65^, which holds that the health-relevant significance of a physical environment derives not from its material attributes but from the affective bonds and place meanings that residents develop through repeated social and cultural engagement. In traditional Dong settlements, where older adults have resided for decades and share deeply rooted collective memories tied to specific spatial settings, such place-based affective bonds are likely to amplify the health-promoting potential of perceived environmental quality, rendering subjective appraisal a more proximate determinant of health than objective spatial configuration. This theoretical account is consistent with Zhang et al.^66^, who found that community cohesion and sense of security mediated built environment-health associations, as well as with the full mediation of OBE by PBE, LA, and SP observed in the present findings. Collectively, these results suggest that in culturally embedded rural settlements, OBE functions as a distal determinant of health whose effects are entirely channeled through perceptual and behavioral intermediaries. This pattern challenges the environmental determinism prevalent in urban built environment research and implies that age-friendly interventions should prioritize the cultivation of environmental awareness and social participation alongside physical infrastructure improvements.

### Roles of leisure activities and social participation

Another core contribution of this study lies in revealing the complex mediating mechanisms of LA and SP between the built environment and older adults’ health. The research confirmed that LA and SP are each significantly and positively associated with health, consistent with extensive prior research^67,68^, and a progressive relationship was identified whereby LA is significantly associated with facilitating SP. Within the cultural context of Dong settlements, many LA, such as neighborly visits, collective trips to markets, and festival celebrations, are inherently embedded in social interactions, serving as important vehicles for maintaining neighborhood relationships. For OBE, both LA and SP served as significant full mediators, with indirect effects of 0.052 (95% CI = [0.006, 0.117]) and 0.057 (95% CI = [0.014, 0.119]) respectively, collectively accounting for the entirety of OBE’s association with health outcomes. For PBE, both mediators operated as significant partial mediators, with indirect effects of 0.115 (95% CI = [0.052, 0.182]) through LA and 0.068 (95% CI = [0.021, 0.125]) through SP, indicating that behavioral engagement constitutes a substantial component of PBE’s total association with health alongside its direct effect. While these behavioral pathways are statistically robust, their modest effect sizes imply that health gains from environmental optimization are likely incremental and should be viewed as supplementary components within a broader health intervention system.

Of particular importance, the chain mediation pathway “PBE → LA → SP → HS” was statistically significant (indirect effect = 0.019, 95% CI = [0.004, 0.037]), whereas the corresponding pathway originating from OBE was not significant (indirect effect = 0.008, 95% CI = [0.000, 0.024]), suggesting that the sequential behavioral mechanism is activated only when the built environment is subjectively perceived rather than objectively characterized. This “perception-behavior-health” associational chain highlights that in traditional “acquaintance societies,” social mechanisms may play more fundamental roles than individualistic behaviors such as independent exercise. This finding provides empirical support for the application of Social Capital Theory in health geography: consistent with Putnam et al.^69^, positively perceived built environments appear to serve as the material foundation for social capital generation, through which LA and SP are transformed into tangible health benefits via emotional support and informational mutual assistance, thereby revealing the underlying mechanism through which the built environment may promote health by cultivating social capital in traditional communities.

### Implications

#### Theoretical implications

This study contributes to theoretical development in two respects. The extension of the “built environment → behavior → health” paradigm to traditional ethnic minority settlements establishes that perceptual mediation and behavioral mechanisms identified in urban contexts retain explanatory power across markedly different socio-spatial configurations, thereby delineating the contextual boundary conditions of established environmental health frameworks. More substantively, the finding that OBE exerts no direct health effect while PBE operates through both direct and indirect pathways suggests that the relative weight of objective versus subjective environmental dimensions is contingent upon cultural context: in collectivist, tradition-based communities where shared spatial meanings are deeply embedded, subjective appraisal and social practice constitute the primary mechanisms through which the built environment becomes health-relevant. This represents a theoretically meaningful refinement rather than mere replication, offering a culturally situated account of how environmental health logic operates beyond the modern urban settings in which it was originally formulated.

### Practical implications

The findings carry direct implications for age-friendly planning in traditional rural settlements, pointing to three interconnected intervention priorities.

Physical renewal strategies should move beyond infrastructure improvement toward activating the perceptual and behavioral pathways through which spatial conditions translate into health outcomes. Preservation of culturally significant spatial elements, including traditional street scales, architectural aesthetics, and shared gathering spaces, provides the material substrate for place attachment and collective identity. Micro-renewal approaches such as optimized paving, lighting, seating, and rest facilities can enhance comfort, safety, and accessibility without compromising cultural character. The use of local materials and traditional craftsmanship further reinforces residents’ sense of belonging.

The planning objective should shift from constructing spaces to supporting behaviors, with spatial design explicitly oriented toward facilitating leisure activities and social participation among older adults. Informal public spaces inherent to traditional villages, including alley entrances, wind-and-rain bridges, opera stages, and drum tower plazas, embody established social functions and should be prioritized for protection and optimization. Design interventions should account for older adults’ walking radii, mobility capacities, and visual accessibility, while small-scale neighborhood spaces such as courtyard gardens and pocket plazas can encourage spontaneous interaction in daily life.

Community development policies should be integrated with spatial interventions to activate the social functions of traditional spaces through institutional and programmatic means. Regular cultural activities such as Dong village council meetings, neighborhood gatherings, and intergenerational folk performances can transform built spaces into venues for social engagement, thereby reinforcing community cohesion and emotional support. A co-creation mechanism involving local governments, research institutions, and community residents ensures that spatial decisions remain responsive to older adults’ living habits and cultural expectations.

Taken together, age-friendly development in traditional settlements is more productively understood as a process of cultural continuity and social vitality revitalization than as material improvement alone, with the ultimate objective of enabling spaces to support health, foster emotional connection, and sustain community life.

### Limitations and future research directions

Although this study has yielded valuable findings, several limitations warrant acknowledgement. First, the cross-sectional design precludes definitive causal inferences. Reverse causality cannot be ruled out: older adults in better health may perceive their environment more favorably, rendering PBE a consequence rather than a determinant of health; similarly, higher baseline health capacity may enable more frequent leisure activities and social participation, inverting the posited mediation pathways. Longitudinal or experimental designs would be necessary to establish temporal precedence. Second, the sample was concentrated in the Dong ethnic region of Western Hunan, potentially limiting generalizability; cross-regional comparative studies are needed to test the model’s broader applicability. Finally, reliance on self-report measures may introduce recall bias and common method variance, and future research should incorporate objective health indicators and time-separated data collection to address this concern.

## Conclusion

Using traditional Dong ethnic settlements in Western Hunan as a case study, this research examined the associations between built environment and older adults’ health through parallel-and-serial mediation models. The findings indicate that objective spatial attributes operate on health exclusively through perceptual and behavioral intermediaries, while perceived built environment exerts both direct and indirect effects via leisure activities, social participation, and their sequential pathway. These results suggest that in traditional rural settlements characterized by acquaintance-based social structures, the health-relevant effects of the built environment are contingent upon residents’ subjective environmental perceptions and sociocultural practices, rather than physical spatial attributes alone. Given the cross-sectional design adopted in this study, the observed patterns reflect associations rather than causal relationships. To establish the directionality among variables, future research could employ longitudinal research designs to further verify the causal pathways.

## Data Availability

The datasets used and analyzed during the current study are available from the corresponding author upon reasonable request.
